# Interrupted time series analysis of children’s blood lead levels: A case study of lead hazard control program in Syracuse, New York

**DOI:** 10.1371/journal.pone.0171778

**Published:** 2017-02-09

**Authors:** Liyang Shao, Lianjun Zhang, Zhen Zhen

**Affiliations:** 1 Department of Forest and Natural Resources Management, State University of New York, College of Environmental Science and Forestry, Syracuse, New York, United States of America; 2 School of Forestry, Northeast Forestry University, Harbin, People’s Republic of China; University of Cincinnati, UNITED STATES

## Abstract

Children’s blood lead concentrations have been closely monitored over the last two decades in the United States. The bio-monitoring surveillance data collected in local agencies reflected the local temporal trends of children’s blood lead levels (BLLs). However, the analysis and modeling of the long-term time series of BLLs have rarely been reported. We attempted to quantify the long-term trends of children’s BLLs in the city of Syracuse, New York and evaluate the impacts of local lead poisoning prevention programs and Lead Hazard Control Program on reducing the children’s BLLs. We applied interrupted time series analysis on the monthly time series of BLLs surveillance data and used ARMA (autoregressive and moving average) models to measure the average children’s blood lead level shift and detect the seasonal pattern change. Our results showed that there were three intervention stages over the past 20 years to reduce children’s BLLs in the city of Syracuse, NY. The average of children’s BLLs was significantly decreased after the interventions, declining from 8.77μg/dL to 3.94μg/dL during1992 to 2011. The seasonal variation diminished over the past decade, but more short term influences were in the variation. The lead hazard control treatment intervention proved effective in reducing the children’s blood lead levels in Syracuse, NY. Also, the reduction of the seasonal variation of children’s BLLs reflected the impacts of the local lead-based paint mitigation program. The replacement of window and door was the major cost of lead house abatement. However, soil lead was not considered a major source of lead hazard in our analysis.

## Introduction

Low lead exposure in early childhood can cause adverse behavioral and development outcomes; as a result, no lead level is considered safe[1]. A number of recent studies [2–5] have reported decrements in IQ and other adverse effects at blood lead levels less than what was previously considered a safe level (10 micrograms per deciliter (μg/dL)). For many years, federal and state governments have devoted considerable effort to limit lead use in the United States. National surveys and state and local surveillance data indicate that children’s blood lead levels (BLLs) have declined in the past decades [6–8]. In 2012, the Center for Disease Control and Prevention (CDC) reduced the BLL at which public health intervention is warranted from 10μg/dL to 5μg/dL which is the 97.5^th^ percentile of BLLs among U.S. children aged 1–5 years old[9].At the national level, 2.5% of children under the age of six have BLLs greater than 5μg/dL (i.e., the elevated BLLs) [9].However, in some inner cities, the proportion of children with BLLs at or above the actionable level of lead poisoning remains significantly high. For example, in the city of Syracuse, NY, a total of 4903 children under the age of 6 years were tested at least once in 2011, and 168 (3.43%) of these children had BLLs ≥ 10μg/dL and 900 (18.36%) children ≥ 5μg/dL.

Over the last two decades, the surveillance data of children’s BLLs have been collected in the city of Syracuse, NY. However, the long-term impacts of the lead hazard control program on observed changes in the trend and seasonal variations in the children’s BLLs are largely unknown. Researchers and policy makers are interested in the long-term trends as well as the temporal or seasonal fluctuations of the children’s BLLs in a region or city. The trends of the children’s BLLs time series can be significantly altered by the governments’ laws and regulations, such as Control of Lead Poisoning-NYS Public Health Law, Title 10 of Article 13;NYS Regulations for Lead Poisoning Prevention and Control-NYCRR title X, Par 67; and Public Health Law Section 2168-Statewide Immunization Registry. Appropriate analysis and modeling, such as interrupted time series analysis, are necessary to investigate the impacts of these laws and regulations on the dynamics of the children’s BLLs. Interrupted time series is a quasi-experiment design, to evaluate the impact of an intervention on its target population when randomization is not possible [10]. In this study we attempted to apply interrupted time series analysis to investigate the changes of children’s BLLs before and after the period of lead-paint mitigation activities in the city of Syracuse, NY. The objectives of the study were: (1) to detect and model the trends and seasonal patterns in the children’s BLLs before and after the intervention; and 2) to evaluate the effectiveness of the Syracuse Lead Program on reducing the children’s BLLs overtime.

## Materials and methods

### Materials

The surveillance data of children’s BLLs in the city of Syracuse, NY were provided by the Onondaga County Health Department (OCHD). All lead testing results are reported to the New York State Department of Health through what is currently called “LeadWeb”. The BLL records included individual child information such as home address, birthday, sample collection date, blood sampling type, blood lead test reading, gender, race, and ethnicity.

The time period of the surveillance data consisted of 237 months from April 1992 to December 2011. There were a total of 83,127 records of children’s blood lead tests from children living within the city of Syracuse, NY. Since the surveillance data included both lead screening and follow up records, the children with elevated BLLs had more test results than other children. Thus, it was not a random sampling procedure. In order to improve the randomness of the samples in children, we selected only the first BLL record in each year for each child tested in that year and ended up with 43,045 records. Note that it requires health care providers to confirm a child's capillary blood lead test result of ≥ 10μg/dL using a venous blood sample. This updates the previous regulation, which required confirmation of capillary test results ≥ 15μg/dL, and is consistent with the national guidelines from CDC to maximize the identification of children with lead poisoning. We then computed the geometric mean of the available children’s BLLs in each month. Thus, we had a total of 237 monthly observations of the children’s BLLs in the time series.

In addition, we collected information on the homes (the children’s BLL ≥10μg/dL) where lead hazard prevention had been completed by the Syracuse Lead Program, including the home’s location, date of project completion, number of units if a multi-family building, total cost of interventions and break-down of costs. We used these data to help evaluate the impact of the lead program on the reduction of the children’s BLLs.

### Methods

In this study, we applied interrupted time series analysis and piecewise regression to evaluate the impacts of the lead exposure in the city of Syracuse, NY. Our analyses included the following steps: (1) an interrupted time series model with three stages of the lead exposure intervention to test if the means of the children’s BLL changed before and after each intervention; (2) a piecewise regression to detect if the slope (trend) of the children’s BLL time series changed before and after each intervention; and (3) segmented time series method to model possible seasonal variations in the children’s BLLs at different intervention stages. All statistical analyses were conducted using SAS 9.3 [11]. The significance level was set at α = 0.05.

#### Interrupted time series analysis

**Fig 1:**
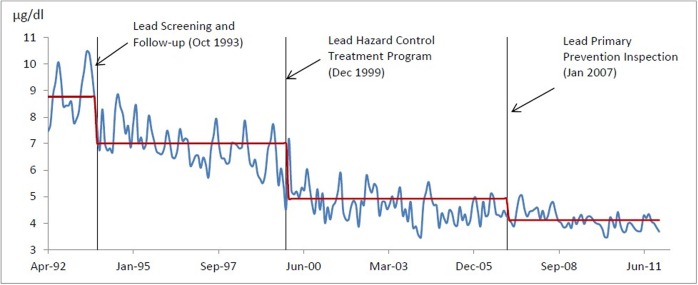
Level (mean) shift of children's BLLs after each intervention in the city of Syracuse, New York.

An interrupted time series analysis model was used to incorporate an underlying disturbance process and a set of interventions [10, 12]. The overall modeling strategy was to obtain the reasonable representations of a deterministic component that describes the intervention and response variable (children’s BLLs) and a stochastic disturbance component [13] that represents the temporal autocorrelation. There were three stages of intervention for the lead exposure prevention over the past 20 years in the city of Syracuse, NY (Fig 1):

- Stage 1. The New York State regulations for reporting the children’s BLLs, lead screening and follow-up, and the role of Onondaga County Health Department Lead Poisoning Prevention Program went into effect in October of 1993 (NYS Regulations for Lead Poisoning Prevention and Control—NYCRR Title X, Part 67 (Volume A-1a)). The environmental assessment and abatement regulation began in January 1995. Those regulations are still in effect.
- Stage 2. The Syracuse Lead Program completed the first lead-paint abatement project in December of 1999 in the city of Syracuse, NY, aimed at the highest risk homes, i.e. those having children with the elevated BLLs (i.e., the children’s BLL ≥10μg/dL). The program received continuous funding from 1999 to 2011 to remove lead in low income housing.
- Stage 3. The lead poisoning primary prevention program started in January of 2007 in the city of Syracuse, NY to conduct inspections and effect the enforcement of identified hazards in properties where no child with an identified elevated lead level (i.e., the children’s BLL ≥10μg/dL) resided. Syracuse Lead Program implemented the lead hazard control treatments to reduce lead paint risks identified during primary prevention inspections.

To capture the serial autocorrelation in the children’s BLLs, an autoregressive and moving average (ARMA) model was specified as part of the time series model. Given the three stages of intervention, the model was expressed as follows [13]:

$$Y_{t}=C+\omega_{1}I_{It}+\omega_{2}I_{2t}+\omega_{3}I_{3t}+N_{t}$$ (1)

where

$$I_{1t}=\left\{ \begin{array}{l} 1,\;t\geq November\;1993\\ 0,\;otherwise \end{array}\right.$$

$$I_{2t}=\left\{ \begin{array}{l} 1,\;t\geq November\;1999\\ 0,\;otherwise \end{array}\right.$$

$$I_{3t}=\left\{ \begin{array}{l} 1,\;t\geq January\;2007\\ 0,\;otherwise \end{array}\right.$$

and *Y*_t_ is the geometric mean of the children’s BLLs, *C* is a constant, *I*_*it*_, (i = 1, 2, and 3) is a binary indicator variable (coded as 0 or 1) that defines the stage of the three interventions, *ω*_*i*_, (i = 1, 2, and 3) is the effect of the interventions. If an intervention is effective, the mean of the children’s BLLs in the post-intervention period is lower than that in the pre-intervention period. Thus, the indicator variable is statistically significant and the coefficient *ω*_*i*_ indicates the magnitude of reduction in the children’s BLLs [13].

The term *N*_*t*_ is called the disturbance (i.e., the model random error) and can be assumed to follow different stochastic process in order to remedy the problem of serially correlated disturbances. The intervention model (Eq 1) was fit to the entire time series of the children’s BLLs. The mode disturbance *N*_*t*_ was assumed to follow the seasonal ARMA (Eq 2), which included the long-term autoregressive parameter *ϕ*, the long-term seasonal autoregressive parameter Ф, the short-term moving-average parameter θ, and the short-term seasonal moving-average parameter Θ. The autocorrelation function (ACF) and partial autocorrelation function (PACF) were used to identify the specification of *N*_*t*_[13]. The seasonal ARMA model can capture the long term influence (AR), short term error (MA), periodic long term influence (ARS), and periodic short term shocks (MAS) as follows:

$$N_{t}=\frac{\theta \left(B\right)\Theta \left(B^{S}\right)}{\phi \left(B\right)\Phi \left(B^{S}\right)}a_{t}$$ (2)

Where *ϕ* is the autoregressive parameter, Ф is the seasonal autoregressive parameter, θ is the moving average parameter, Θ is the seasonal moving average parameter, s is the number of time periods, a_t_ is i.i.d. as normal *N*(0, $\sigma_{a}^{2}$).

#### Piecewise regression

We were also interested in the reduction rate of the children’s BLLs in each intervention stage. However, if a regression model was fit separately to each section of the time series split by the three interventions the four regression lines would not meet at the intervention points [14]. In order to obtain a continuous trend across the entire time seris piecewise regression was used to link the four linear models, which were connected at the three knots (intervention points). The slope coefficient of each regression model will indicate the trend change or reduction rate of the children’s BLLs during each time period. Let T be the time period from April 1992 to December 2011, and t be a time index from 1 to 237 months. Thus, the three interventions were expressed as T = October 1993, t = 19; T = December 1999, t = 93; and T = January 2007, t = 178. Then, four dummy variables (T_1t_, T_2t_, T_3t_, and T_4t_) were defined as follows:

$$T_{1t}=t$$

$$T_{2t}=t-19\;if\;t\geq 19,\;T_{2t}=0\;otherwise$$

$$T_{3t}=t-93\;if\;t\geq 93,\;T_{3t}=0\;otherwise$$

$$T_{4t}=t-178\;if\;t\geq 178,\;T_{4t}=0\;otherwise$$

Thus, the piecewise regression model was specified according to Johnston and DiNardo [14]:

$$Y_{t}=\alpha_{1}+\delta_{1}T_{1t}+\delta_{2}T_{2t}+\delta_{3}T_{3t}+\delta_{4}T_{4t}+\varepsilon_{t}$$ (3)

The intercept and slope coefficients of the linear model can be obtained for each section of the time series as follows:

- T < Nov. 1993: *Y*_*t*_ = *α*_1_ + *β*_1_*T* + μ_*t*_
                                  where *α*_1_ = *α*_1_ and β_1_ = *δ*_1_
- Nov. 1993 ≤ T < Dec. 1999: *Y*_*t*_ = *α*_2_ + *β*_2_*T* + μ_*t*_
                                  where α_2_ = *α*_1_ – *δ*_2_ ∙ 19 and β_2_ = *δ*_1_ + *δ*_2_
- Dec. 1999 ≤ T < Jan. 2007: *Y*_*t*_ = *α*_3_ + *β*_3_*T* + μ_*t*_
                                  where α_3_ = *α*_2_ – *δ*_3_ ∙ 93 and β_3_ = *δ*_1_ + *δ*_2_ + *δ*_3_
- Jan. 2007 ≤ T: *Y*_*t*_ = *α*_4_ + *β*_4_*T* + μ_*t*_
                                  where α_4_ = *α*_3_ – *δ*_4_ ∙ 178 and β_4_ = *δ*_1_ + *δ*_2_ + *δ*_3_ + *δ*_4_

#### Segmented time series

The previous intervention model (Eq 1) included the three interventions so that the model coefficients reflected the joint effects of the intervention stages, in which only one stochastic process *N*_*t*_ was specified. From the time series plot variation, we assumed that the stochastic process of the series was changed because of three intervention activities in different time periods [15]. To examine the hypothesis, the entire time series of the children’s BLLs was divided into three segments, each included only one intervention (one indicator variable). Then, three intervention models (Eqs 4–6) were used to estimate the parameters of the disturbance term ARMA(1,1)×(1,1)_12_ and detect the pattern changes of the AR and MA components. The models were specified as follows:

1. Time period from April 1992 to December 1999:
  $$Y_{t}=C+\omega_{1}I_{It}+\frac{\theta \left(B\right)\Theta \left(B^{S}\right)}{\phi \left(B\right)\Phi \left(B^{S}\right)}a_{t}$$ (4)
  $$I_{1t}=\left\{ \begin{array}{l} 1,\;t\geq November\;1993\\ 0,\;otherwise \end{array}\right.$$
2. Time period from November 1993 to January 2007:
  $$Y_{t}=C+\omega_{2}I_{2t}+\frac{\theta \left(B\right)\Theta \left(B^{S}\right)}{\phi \left(B\right)\Phi \left(B^{S}\right)}a_{t}$$ (5)
  $$I_{2t}=\left\{ \begin{array}{l} 1,\;t\geq November\;1999\\ 0,\;otherwise \end{array}\right.$$
3. Time period from December 1999 to December 2011:
  $$Y_{t}=C+\omega_{3}I_{3t}+\frac{\theta \left(B\right)\Theta \left(B^{S}\right)}{\phi \left(B\right)\Phi \left(B^{S}\right)}a_{t}$$ (6)
  $$I_{3t}=\left\{ \begin{array}{l} 1,\;t\geq January2007\\ 0,\;otherwise \end{array}\right.$$

## Results

### Level (mean) shift

First, this study investigated the level (mean) shift of the children’s BLLs after each intervention in the city of Syracuse, New York. It was clear that the time series shows a downward trend and a marked seasonal pattern (Fig 1). The mean of the children’s BLLs decreased from around 9μg/dL to 7μg/dL after lead screening and follow-up (October 1993), and reduced to 5μg/dL after lead hazard control treatment program conducted (December 1999), and then dropped to 4μg/dL after lead primary prevention inspection carried out (January 2007). Thus, the primary prevention inspection and lead hazard control treatment had a significant impact on reducing the lead-based paint exposure for children in the city of Syracuse, New York.

The time series analysis indicated that the disturbance term of the intervention model was ARMA(1,1)×(1,1)_12_, indicating the 12-month lag and 1-month lag were significant. The maximum likelihood method was used to estimate the model parameters of the intervention and the results were shown in Table 1. It was evident that all model parameters were statistically significant at the significance level α = 0.05, except the first-order moving-average parameterθ_1_ (p-value = 0.3641). Both seasonal parametersΘ_1_ (MA) andФ_1_ (AR) were close to 1, indicating strong seasonal variation was both long-term and short-term. While the short-term influenceθ_1_ was not significant, the long-term autoregressive parameter *ϕ*_1_ = 0.6189 and was statistically significant. The autocorrelation function (ACF) of the model residuals indicated that the specification of the model was adequate and the residual plot of the residuals did not show autocorrelation.

**Table 1:** Coefficient estimation of the intervention model ARMA(1,1)×(1,1)_12_ (Eq 1).

Parameter	Coefficient	Standard error	t	p-value	Lag
**C**	8.5930	0.2770	31.02	< .0001^a^	0
**θ**_**1**_	0.1088	0.1199	0.91	0.3641	1
**Θ**_**1**_	0.8898	0.0763	11.66	< .0001^a^	12
***ϕ***_**1**_	0.6189	0.0952	6.50	< .0001^a^	1
**Φ**_**1**_	0.9762	0.0279	35.05	< .0001^a^	12
**ω**_**1**_	-1.7604	0.2570	-6.85	< .0001^a^	0
**ω**_**2**_	-2.0905	0.1835	-11.39	< .0001^a^	0
**ω**_**3**_	-0.5613	0.1864	-3.01	0.0026^a^	0

^a^Model parameter was statistically significant at the significance level α = 0.05.

The model coefficients of the three binary indicators of interventions were all statistically significant, meaning that the level (mean) of the children’s BLLs had a significant shift or decline after each lead intervention. For example, the estimated ω_1_ = -1.7604 indicated that after the first intervention stage in October 1993 (i.e., NY State regulations) the children’s BLLs declined1.76μg/dL on average. The estimated ω_2_ = -2.0905 demonstrated that after the second intervention stage in December 1999 (i.e., HUD funded lead hazard control treatment program) the average children’s BLLs was reduced 2.09μg/dL, and the estimated ω_3_ = -0.5613 showed that after the third intervention stage in January 2007 (i.e., the NYSDOD Childhood Lead Primary Prevention Program) the average children’s BLLs declined 0.56μg/dL.

### Trend change

Piecewise regression was used to estimate the regression models for the four sections of time series of the children’s BLLs split by the three stages of intervention and the four regression lines were connected at the three knots (intervention points). The slope coefficient of each regression model indicated the trend change or reduction rate of the children’s BLLs during each time period. The downward trend became flat after each intervention, and it indicated BLLs stable declined in each time period. The piecewise regression (Eq 3) showed in Table 2 and the four regression models were illustrated in Table 3. It seemed that the rate changes of the children’s BLLs in the first (slope = -0.03229) and second (slope = -0.03769) time periods were similar, and the difference between the two slopes was not significant because the p-value for testing the null hypothesis of δ_2_ = 0 was 0.7688. However, the slope (-0.01514) of the third time period was significantly different from the previous ones because the p-value for testing the null hypothesis of δ_3_ = 0 was < 0.0001, indicating the reduction rate of the children’s BLLs between December 1999 and January 2007 was significantly smaller than those before December 1999. Again, the slope (-0.0042) of the fourth time period was significantly different from that of the third time period, it indicating that the reduction rate of the children’s BLLs became smaller after January 2007 (Tables 2 and 3).

**Table 2:** Coefficient estimation and test of the piecewise regression (Eq 3).

Parameter	Coefficient	Standard error	t	p-value
α_1_	8.8654	0.2693	32.92	<0.0001^a^
δ_1_	-0.03229	0.0170	-1.90	0.0586
δ_2_	-0.00540	0.0183	-0.29	0.7688
δ_3_	0.02255	0.0039	5.84	<0.0001^a^
δ_4_	0.01094	0.0049	2.26	0.0249^a^

^a^Model parameter was statistically significant at the significance level α = 0.05.

**Table 3:** Regression models for the four time periods derived from the piecewise regression.

Segment	Time Period	Regression Model
1	T < Nov. 1993	Y_t_ = 8.8654–0.03229·T
2	Nov. 1993 ≤ T < Dec. 1999	Y_t_ = 8.9680–0.03769·T
3	Dec. 1999 ≤ T < Jan. 2007	Y_t_ = 6.8709–0.01514·T
4	Jan. 2007 ≤ T	Y_t_ = 4.9236–0.00420·T

### Seasonal pattern change

We suspected that the HUD funded Lead Hazard Control Treatment might change the seasonal or periodic pattern of the children’s BLLs. The model fitting results (Table 4) show otherwise. Time period 1 (April 1992 to December 1999) included the first intervention in October 1993 (i.e., NY State regulations). Both lag 1 MA parameter θ_1_ and lag 12 MA parameters Θ_1_ were not statistically significant (p-values > 0.05), indicating the short-term influence and seasonality were not strong. But the lag 1 AR parameters *ϕ*_1_ was very significant (p-value < 0.0001) which indicated a long term influence. Time period 2 (October 1993 to January 2007) included the second intervention in December 1999 (i.e., lead hazard control treatment program). The model fitting resulted in all model parameters being statistically significant, except the lag 1 MA parameterθ_1_ (p-value = 0.3760). Time period 3 (December 1999 to December 2011) included the third intervention in January 2007 (i.e., the lead poisoning primary prevention program). The model fitting results showed that all lag 1 and lag 12 AR and MA parameters were significant (p-value < 0.001). It was evident that the short-term influence became more important over time (Table 4). In addition, the policy effect before and after the intervention in each time segment, was estimated by the magnitude of the coefficient estimate of the indicator variable in each intervention model, which indicated the reduction of the children’s BLLs after each intervention. Compared to interrupted time series analysis, segmented time series focused on the reduction in a fixed time period. For example, time period 2 had the largest reduction level 2.1357μg/dL after 7 years lead hazard control treatment in the city of Syracuse, NY (Table 4).

**Table 4:** Coefficient estimation and test of the segmented intervention models with ARMA(1,1)×(1,1)_12_.

Parameter	Coefficient	Standard Error	t	p-value	Lag
Time Period 1: April 1992—December 1999					
C	8.4150	0.3341	25.19	< .0001^a^	0
θ_1_	-0.0128	0.1791	-0.07	0.9432	1
Θ_1_	-0.1152	0.2613	-0.44	0.6594	12
***ϕ***_**1**_	0.5979	0.1450	4.12	< .0001^a^	1
Φ_1_	0.3948	0.2310	1.71	0.0874	12
ω_1_	-1.5513	0.3284	-4.72	< .0001^a^	0
σ = 0.5388					
Time Period 2: November 1993—January 2007					
C	6.8501	0.2082	32.90	< .0001^a^	0
θ_1_	0.1351	0.1526	0.89	0.3760	1
Θ_1_	0.9805	0.0162	60.59	< .0001^a^	12
***ϕ***_**1**_	0.6099	0.1226	4.97	< .0001^a^	1
Φ_1_	0.9988	0.0009	1136.14	< .0001^a^	12
ω_2_	-2.1357	0.1847	-11.56	< .0001^a^	0
σ = 0.5468					
Time Period 3: December 1999—December 2011					
C	4.6964	0.2331	20.15	< .0001^a^	0
θ_1_	0.4379	0.1244	3.52	0.0004^a^	1
Θ_1_	0.9788	0.0206	47.47	< .0001^a^	12
***ϕ***_**1**_	0.8307	0.0740	11.23	< .0001^a^	1
Φ_1_	0.9987	0.0013	777.29	< .0001^a^	12
ω_3_	-0.5461	0.1975	-2.77	0.0057^a^	0
σ = 0.4217					

^a^Model parameter was statistically significant at the significance level α = 0.05.

## Discussion

### The trend and pattern changes of children’s BLLs

Our study used the available surveillance data of children’s BLLs to investigate the long-term trend and seasonality variation over the last 20 years in the city of Syracuse, New York. The average children’s BLLs decreased from 8.77μg/dL in 1992 to 3.94μg/dL in 2011 for children under six years old, which was a 55% decline. The percentage of children with the BLLs ≥ 10μg/dL reduced from 44.08% to 3.43% (a 92% decline), while the percentage of children with BLLs ≥ 5μg/dL reduced from 97.79% to 18.36% (81% decline). However, although the average children’s BLLs had decreased dramatically in the city of Syracuse, more children with elevated BLL in Syracuse than many other cities in US [7].

The interrupted time series models indicated that the average children’s BLLs was significantly reduced in the three post-intervention periods, but at different declining rates. For example, there was a 1.55μg/dL decline over the 6-year time period (0.26μg/dL per year) after the first intervention stage. When the HUD funded lead hazard control treatment program started in the city of Syracuse, the average children’s BLLs reduced 2.14μg/dl over the 7-year periods (0.31μg/dL per year). In the last intervention period, there was a 0.55μg/dl decline over the 5-year period (0.11μg/dL per year). Thus, the three post-intervention periods had significant impacts on reducing the lead-based paint exposure for children in the city of Syracuse, New York.

In this study, we observed that the city of Syracuse had a distinctive periodic pattern before the HUD funded lead hazard control treatment program. The peak BLLs months were in summer (particularly June, July, and August) and the lowest BLLs months were in winter from year to year, but the amplitude of the seasonal variation decreased over time. However, the lead hazard control treatment program aimed at reducing environmental lead hazard exposure made the regular seasonal cycle of children’s BLLs more complicated. The peak BLLs month were not always in the summer time, it also related to the intensity of the lead abatement work. From April 1992 to December 1999, there was no lead-based paint mitigation and the only intervention was the NY State health laws and regulations. The segmented time series model in this time period indicated that there was only long-term regular (AR) and long-term seasonal influence in the random structure. The AR components described the stochastic process which the current value was dependent on its past values, and they were considered the infinite impulse and perceived as the long-term influence. The long-term influence could be conceptualized as the natural environment influence, such as temperature, precipitation, dust, or children’s behavioral patterns (e.g., during summer, children play outside and are more likely to be exposed to soil lead).

On the other hand, during the period of December 1999 to December 2011, the Syracuse Lead Program completed lead hazard control treatment projects on more than 1400 home units. The segmented time series model showed that both long-term regular, seasonal pattern and short-term regular, seasonal pattern were significant (Table 4), indicating the mixed influences of both long-term and short-term influence. This process could be conceptualized as long-term natural influence with short-term lead abatement project impacts. It was evident that the lead hazard control treatment program had short-term impacts on children’s BLLs and the natural periodic seasonal pattern had been disturbed by the human lead paint mitigation activities, which induced a more complicated variation pattern for the children’s BLLs in the city of Syracuse, New York.

Past studies had reported the seasonality of children’s BLLs as regular periodic cycles in the city of Syracuse, New York [16], as well as in other cities [17–19]. It had been described as the cycle with a peak in August and the lowest point in March [17]. Some studies recorded that this seasonal variation could be predicted by temperature, soil moisture, and dust variables [18–19]. However, this study provided evidence indicating that the seasonality of the BLLs was not much caused by lead in soil or the dust from soil, rather than it was mainly influenced by lead-based paint exposure. In summer months, the inner city windows with paint were used for natural ventilation, and more lead paint was eroded off the windows and entered the indoor dust pool. In the winter time, the windows stayed closed in Syracuse, because it was too cold. The lead hazard control treatment reduced the lead paint exposure from windows, doors and some other parts in the houses. Thus, the seasonality of children’s blood lead levels diminished as well.

### The impact of lead hazard control treatment

**Fig 2:**
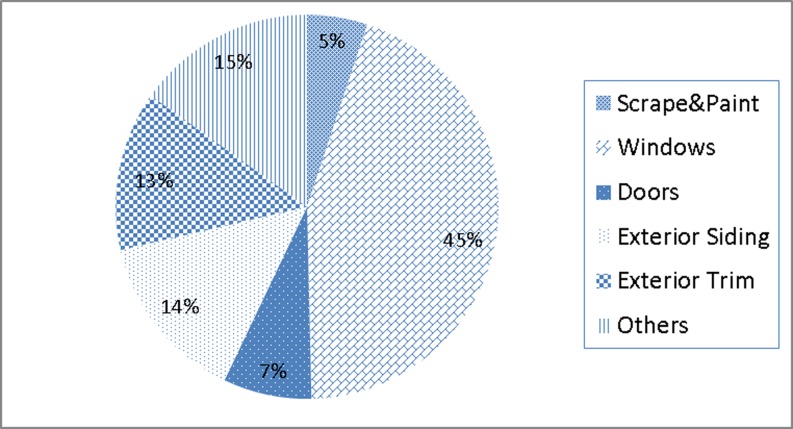
Cost break down of 450 completed house units from 1999 to 2006 in the city of Syracuse, New York.

The lead hazard control treatment program removed lead based paint by replacing new windows, doors and siding in the houses. The most cost of the lead hazard control treatment was for the window replacement (45% of the total cost), followed by exterior siding and exterior trim (Fig 2). Past research showed that 12 years after window replacement and residential lead paint hazard control the homes had 41% less interior floor dust and 51% lower dust lead [20]. Our study provided the quantitative proof that after the efforts of the lead hazard control treatment the average of children’s BLLs was significantly reduced by 50% in ten years on average, and the lead hazard control program (the second stage) had the largest reduction level, reduction level of 2.14μg/dL (Table 4).

**Fig 3:**
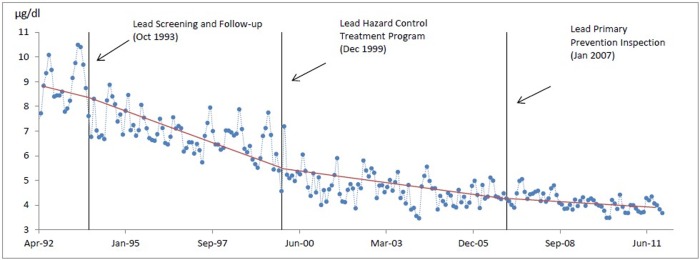
Trend change of children's BLLs after each intervention in the city of Syracuse, New York.

The blood lead level reduction rate was decreasing over time, and the piecewise regression showed that the absolute value of the slopes became smaller (Table 3 and Fig 3). Since what year the average of the children’s BLLs remained at a relatively stable amount of 4μg/dl in the city of Syracuse. This was similar to other effectiveness research [21] who found post-intervention geometric mean BLLs for children were approximately 3.5μg/dL. This may also indicated the difficulty in lowering BLLs much below the threshold. This evidence might indicate that the lead paint abatement activities alone may not be able to further reduce the children’s BLLs, since the children were potentially exposed to other environmental lead sources such as lead contaminated food, lead in drinking water, and etc. This would require more public health laws enforcement efforts to identify other various sources of lead risks. The CDC recently lowered the threshold values of “level of concern” from 10μg/dL to 5μg/dL to enforce the risk reduction activities.

### Limitations

Since children’s lead poisoning was a prevalent environmental epidemiology in USA, similar interventions had been applied in the entire country. It was impossible to find similar high risk communities which were not under the same or similar public health laws and regulations and without lead hazard control treatment. Therefore, non-experiment design studies had to be conducted. Thus, the causal relationship from the interrupted time-series was limited. First, we didn’t have sufficient observations available before the first intervention to draw the conclusion that the first intervention caused the reduction of the children’s BLLs from 1994 to 1999, and the federal laws and regulations for eliminating lead use could be a latent factor to the BLLs decline in this period. Second, the effect of the lead hazard control treatment was difficult to separate from the impacts of public awareness, so we calculate the level of reduction also including the impacts of public health laws. The other underlying problem was that the sample size changed over time. During 1995 to 2004, the number of children taking the BLLs tests was less than the other time periods, which might lead to a larger variance in the average children’s BLLs.

## Conclusion

Children’s BLLs have reduced continuously in the past two decades. The public health laws enforcing lead screening, follow-up and environmental assessment and abatement have been effective in reducing BLLs. Lead hazard control treatments have also been effective in reducing children’s BLLs. However, abatement activities changed the seasonal pattern cycle in monthly BLLs, and made the seasonal variation more complex. Once children’s BLLs reached a low level, aggressive lead paint abatement appeared to reach a point beyond which it did not contribute further to significant BLL reductions.

## Supporting information

S1 File The data used for fitting the time series models in the study. (XLSX)
